# The anti-fibrotic agent pirfenidone synergizes with cisplatin in killing tumor cells and cancer-associated fibroblasts

**DOI:** 10.1186/s12885-016-2162-z

**Published:** 2016-03-02

**Authors:** Melanie Mediavilla-Varela, Kingsley Boateng, David Noyes, Scott J. Antonia

**Affiliations:** Department of Immunology, H. Lee Moffitt Cancer Center and Research Institute, Tampa, FL 33612 USA; Thoracic Oncology Department, H. Lee Moffitt Cancer Center and Research Institute, Tampa, FL 33612 USA

**Keywords:** Pirfenidone, Cisplatin, Non-small cell lung cancer, Cancer associated fibroblasts

## Abstract

**Background:**

Anti-fibrotic drugs such as pirfenidone have been developed for the treatment of idiopathic pulmonary fibrosis. Because activated fibroblasts in inflammatory conditions have similar characteristics as cancer-associated fibroblasts (CAFs) and CAFs contribute actively to the malignant phenotype, we believe that anti-fibrotic drugs have the potential to be repurposed as anti-cancer drugs.

**Methods:**

The effects of pirfenidone alone and in combination with cisplatin on human patient-derived CAF cell lines and non-small cell lung cancer (NSCLC) cell lines were examined. The impact on cell death in vitro as well as tumor growth in a mouse model was determined. Annexin V/PI staining and Western blot analysis were used to characterize cell death. Synergy was assessed with the combination index method using Calcusyn software.

**Results:**

Pirfenidone alone induced apoptotic cell death in lung CAFs at a high concentration (1.5 mg/mL). However, co-culture in vitro experiments and co-implantation in vivo experiments showed that the combination of low doses of cisplatin (10 μM) and low doses of pirfenidone (0.5 mg/mL), in both CAFs and tumors, lead to increased cell death and decreased tumor progression, respectively. Furthermore, the combination of cisplatin and pirfenidone in NSCLC cells (A549 and H157 cells) leads to increased apoptosis and synergistic cell death.

**Conclusions:**

Our studies reveal for the first time that the combination of cisplatin and pirfenidone is active in preclinical models of NSCLC and therefore may be a new therapeutic approach in this disease.

**Electronic supplementary material:**

The online version of this article (doi:10.1186/s12885-016-2162-z) contains supplementary material, which is available to authorized users.

## Background

Cellular transformation to neoplasm, tumor formation, and progression require an inherent crosstalk between the tumor microenvironment and the tumor. The tumor microenvironment or tumor stroma comprises various cell types, including cancer-associated fibroblasts (CAFs), immune cells, endothelial cells, and mesenchymal cells. In this heterogeneous environment, CAFs are the most prominent cell type and are known to be key contributors to tumor progression, invasion, and metastasis [1]. In addition, functionally, they are known to secrete extracellular matrix proteins (e.g., collagen I, III, IV), chemokines (e.g., interleukin 6, CXCL8, CXCL12), cytokines (e.g., interleukin 6 and interleukin 8), and tumor growth factors (e.g., vascular endothelial-derived growth factor, transforming growth factor beta (TGFβ), hepatocyte growth factor, epidermal growth factor, and fibroblast growth factor] [2, 3]. Contact between CAFs and tumor cells have shown increased tumor cell survival via the activation of anti-apoptotic pathways or by induction of the epithelial to mesenchymal transition in melanoma and non-small cell lung cancer (NSCLC) [2, 4–6]. Therefore, a combined therapy that targets both the tumor cell and the CAFs might lead to novel therapies in cancer.

Pirfenidone [5-methyl-1-phenyl-2(1*H*)-pyridone] is a well-recognized anti-fibrotic compound shown to be effective in various in vivo models [7–9] and in clinical trials [10]. In addition, it is endowed with anti-proliferative effects in a variety of cells, spanning from human Tenon fibroblasts [11], cardiac fibroblasts [12], leiomyoma cells [13], lung fibroblasts [14], pancreatic stellate cells, and pancreatic cancer cells [15]. Furthermore, it has been shown that pirfenidone inhibits proliferation, migration, and epithelial-mesenchymal transition of a human epithelial cell line [16], disrupted tumor-stromal interactions in pancreatic cancer [15], and inhibited TGFβ1-induced overexpression of collagen type I in A549 cells [17]. In 2011, pirfenidone was the first drug approved in Europe for the treatment of idiopathic pulmonary fibrosis [18] following its approval in the United States in 2014. Various groups have shown changes in different cytokines and growth factors such as platelet-derived growth factor-A, hepatocyte growth factor, and periostin in pancreatic stellate cells [15], heat shock protein 47 in lung cancer cells [17], TGFβ1 in cardiac fibroblasts [12], and COX-2 and PGE2 in orbital fibroblasts [19]; however, the mechanism by which it exerts its effects is yet to be elucidated.

Cisplatin is a platinum-based chemotherapeutic drug that has been used as the standard of care in late stages of NSCLC in combination with paclitaxel, vinorelbine, gemcitabine, docetaxel, pemetrexed, or irinotecan [20]. Although the combination of cisplatin and other chemotherapeutic drugs has been successful in increasing the survival rate in some patients, the overall response rate remains partial and many do not respond to this combination [20, 21]. Cisplatin is known to intercalate DNA by the formation of cross-links. The damage done by cisplatin leads to inhibition of DNA replication followed by apoptosis or necrosis [20, 22]. Studies have extensively shown the effects of cisplatin in tumor cells, but only a small percentage of studies interrogate its effects on CAFs. Sonnenberg et al. [23] was the first to show that CAFs resected from breast and lung patients had a high variable sensitivity to cisplatin treatment comparable to that of tumor cell lines. Moreover, when co-cultured, they observed that lung cancer cells were more sensitive to cisplatin than CAFs.

Here, we further characterized the effects of pirfenidone in the tumor microenvironment. In this study, we demonstrated that the combination of pirfenidone and cisplatin suppressed the proliferation of NSCLC cells and CAFs. In addition, we examined the effect of pirfenidone and cisplatin in a co-culture model and in an in vivo model using both NSCLC cells and CAFs derived from lung tumor and showed that the combination of pirfenidone and cisplatin has a detrimental effect in both cell populations by the activation of the apoptotic machinery.

## Methods

### Cell culture and reagents

Primary human fibroblasts were isolated from portions of lung tumors resected from adult patients for clinically indicated reasons. Patients had provided written informed consent to allow use of tumor samples. Our study received approval from University of South Florida Internal Review Board (Pro00015693). The tumors were mechanically and enzymatically (collagenase, protease, and DNase) digested, and the cells were cultured in RPMI, 10 % FBS, PenStrep (Life Technologies), and L-glutamine at 37 °C. After 1 week of culture, tumor and immune cells died; however, the CAFs proliferated vigorously and survived for greater than 15 passages. To verify whether cells were actually CAFs, an α-smooth muscle actin Western blot analysis was performed, as well as a flow cytometric analysis after staining with anti-FAP [24]. A549, H157, H2122, H358, H1299, H23 and PC9 cells were purchased from American Type Culture Collection (Manassas, VA) and cultured in RPMI, 10 % FBS, PenStrep, and L-glutamine at 37 °C. Pirfenidone was purchased from TCI America, and cisplatin was obtained from TEVA.

### Morphologic analysis

To examine the morphology of cultured cells after treatment with pirfenidone, 3 × 10^5^ CAF cells/well were seeded in a 6-well culture plate in RPMI. After 24 h, the cells were treated with 1.5 mg/mL of pirfenidone or vehicle control for 48 h. Pictures were taken under a brightfield light with an automated Zeiss Observer Z.1 inverted microscope as described before [24].

### Annexin V/PI analysis

To examine apoptotic cell death, 1.5 × 10^5^ cells/well CAFs or NSCLC cells were seeded into 6-well culture plate in RPMI. After 24 h the cells were treated with pirfenidone at either 0.5 mg/mL or 1.5 mg/mL, 10 μM cisplatin, or vehicle control. Supernatant and cells were collected 24 and 48 h later. The adherent cells were removed from the plate using 250 μL trypsin (Invitrogen) and allowed to rest in complete media for 15 min. Annexin V/PI analysis was performed as described before [24].

### Cell viability assay

The CellTiter 96® AQ_ueous_ One Solution cell proliferation assay (MTS, Promega) was used to examine cell viability. Cells were seeded into 96-well plates at 5 × 10^3^ cells/well. They were pretreated with U0126 at 10 μM (Cell Signaling; 9903) and then treated with escalating doses of pirfenidone or with 0.5 mg/mL of pirfenidone and 10 μM of cisplatin for 72 h. After the treatment period, 20 μL of the MTS solution was added and incubated at 37 °C for 1 h. Plates were read at 490 nm in a BioTek EL808 microplate reader. Treatments were compared to their vehicle control.

### Caspase 3/7 activity assay

The CellPlayer 96-well kinetic caspase 3/7 reagent (Essen Bioscience) was used to assess caspase 3/7 activity. CAF cells were seeded in a 96-well plate at 5 × 10^3^ cells/well. They were pre-treated with Z-VAD.fmk (50 μM) and then treated with pirfenidone (1.5 mg/mL) for 48 h. After treatment, the CellPlayer 96-well kinetic caspase 3/7 reagent was added to the cells at a final concentration of 5 μM. The caspase 3/7 activity assay was performed as described before [24].

### TGFβ1 ELISA

TGFβ1 secretion by CAFs was determined. CAFs were seeded in 6-well plates at 1.5 × 10^5^ cells/well. After 24 h, the cells were treated with pirfenidone (1.5 mg/mL) or control agent for 72 h. The supernatant was collected and a TGFβ1 ELISA (Roche) was performed per manufacturer’s instructions.

### Synergy proliferation assay

The activity levels of drugs alone and in combination were determined by a high-throughput CellTiter-Blue (Promega) cell viability assay. Cells (1.2–2 × 10^3^) were plated in each well of 384-well plates using a Precision XS liquid handling station (Bio-Tek Instruments, Inc.) and incubated overnight. A liquid handling station was used to serially dilute all drugs in media, and 5 μL were added to 4 replicate wells and an additional 4 control wells received a diluent control without drug. At the end of the incubation period with drugs, 5 μL of CellTiter-Blue reagent were added to each well. The fluorescence of the product of viable cells was measured with a Synergy 4 microplate reader (Bio-Tek Instruments, Inc). We determined IC50 values using a sigmoidal equilibrium model regression and XLfit version 5.2 (ID Business Solutions Ltd.). The IC50 values obtained from single-drug cell viability assays were used to design subsequent drug combination experiments.

### Synergy analysis

The effect of drug combination was evaluated using Calcusyn software (Biosoft). The synergy experiment was performed as described above, and the results were analyzed for synergistic, additive, or antagonistic effects using the combination index (CI) method developed by Chou and Talalay [25], with CI < 1, CI = 1, and CI > 1 indicating synergism, additive effects, and antagonism, respectively. A confidence interval of 0.1–0.3 meant strong synergism, 0.3–0.7 indicated synergism, 0.7–0.85 indicated moderate synergism, 0.85–0.90 indicated slight synergism, and 0.90–1.10 indicated nearly additive.

### Co-culture model

A549 cells were stained using Cell Tracker Violet (Invitrogen) per manufacturer’s instructions. Briefly, in a 100-mm dish, stained A549 cells and unstained CAFs were co-seeded at 2.5 × 10^5^ cells/plate. After 24 h, the cells were treated and then harvested after 48 h of treatment. Annexin V/PI analysis was performed as described before on each cell line, which was determined by their Cell Tracker Violet status, positive (A549) or negative (CAF) to the stain.

### Mouse model

A549 cells (4 × 10^6^) and CAFs (4 × 10^6^) were injected subcutaneously into 4- to 6-week-old athymic nude mice (NCI). When tumors were palpable, mice were randomly allocated into 4 groups and treated with daily intraperitoneal injections of (1) control vehicle (H_2_O); (2) pirfenidone alone (200 mg/kg; dissolved in H_2_O) for 27 days; (3) cisplatin alone (5 mg/kg) on days 0, 3, 6, 21, 24, and 27; or 4) pirfenidone and cisplatin as described above. The experiment was terminated when tumors reached 200 mm^2^ or became ulcerated.

### Ethics and permissions

Animal experiments were performed according to a protocol approved by the Institutional Animal Care and Use Committee of the University of South Florida.

### Western blots

Whole cell lysates were collected in 1X CHAPS buffer (Cell Signaling), from pirfenidone-treated A549 cells as well as a human CAF line. Protein concentrations were quantified using the Bio-Rad protein assay dye. Equal amounts of protein (40 μg) were loaded into the wells of a 10 % SDS-PAGE gel and resolved at 100 V for 90 min. Proteins were then transferred to a PVDF membrane, blocked, and then probed for PARP (Cell Signaling; 9542) at 1:2000, phospho-44/42 MAPK (Erk1/2) (Cell Signaling; 4370) at 1:2000, p44/42 MAPK (Erk1/2) (Cell Signaling; 9102) at 1:2000, phospho-Akt (Cell Signaling; 9271) at 1:2000, and Akt (Cell Signaling; 4691) at 1:2000, with an overnight incubation at 4 °C. GAPDH was used at 1:2000 concentration for 30 min at room temperature (Cell Signaling; 2118S). All of the primary antibodies were incubated afterward with anti-rabbit IgG horseradish peroxidase (Cell Signaling; 7074) at 1:2000 for 30 min at room temperature.

### Statistical analysis

Data are shown as means ± SE. Statistical calculations were performed using Student *t* test. Statistical significance was accepted as *P* values less than 0.05.

## Results

### Pirfenidone induces apoptosis in CAFs

**Fig. 1:**
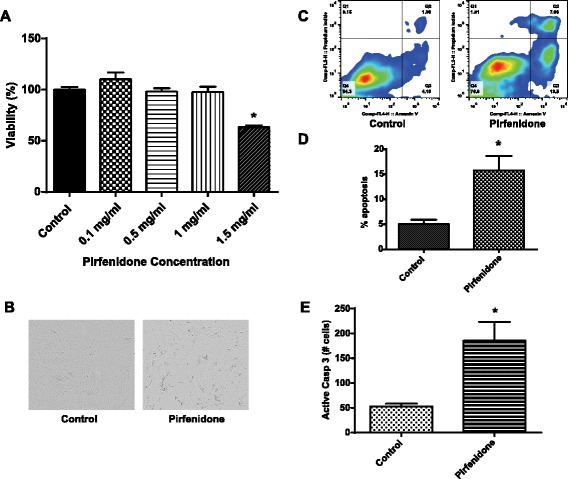
**a** MTS assay showing the viability of lung CAFs after 72-h treatment with varying doses of pirfenidone. **b** Morphological analysis after 72 h of treatment with 1.5 mg/mL pirfenidone shows decreased cell numbers versus control. **c** Representative annexin V/PI flow cytometric analyses in lung CAFs after 48 h of treatment showing increased apoptosis in pirfenidone (1.5 mg/mL)-treated lung CAFs versus control. **d** Average of 3 independent annexin V/PI experiments in lung CAFs. **e** Active caspase 3/7 assay showing increased active caspase 3/7 in lung CAFs after 48 h of treatment with pirfenidone (1.5 mg/mL). *P < 0.05

Pirfenidone has been reported to have inhibitory effects on fibroblast proliferation [7, 11, 12, 14]. However, the effects of pirfenidone in lung CAFs have yet to be explored. To determine whether pirfenidone has any effect on lung CAFs, a proliferation assay was performed with varying concentrations of the drug. Figure 1a shows that high concentrations of pirfenidone (1.5 mg/mL) result in a 40 % decrease in lung CAF proliferation, which was not observed at any other concentration. A morphological analysis (Fig. 1b) also illustrates the effects of the drug on decreasing the number of lung CAFs. To characterize the effect of pirfenidone on lung CAFs, we performed various apoptotic assays. An annexin V/PI analysis (Fig. 1c) was performed on lung CAFs treated with pirfenidone (1.5 mg/mL). The results indicated an increase in early apoptosis (annexin V+/PI-; lower right panel) and late apoptosis (annexin V+/PI+; upper right panel) after 72 h of treatment. In 3 independent experiments, the average percentage of early apoptotic cell death in the treated cells (15.8 %) was statistically significant (*P* = 0.02) when compared to its untreated control (5 %) (Fig. 1d). In addition, in a caspase 3/7 analysis performed using the CellPlayer 96-well kinetic caspase 3/7 reagent, an almost 3-fold increase in caspase 3/7 was observed in CAFs treated with pirfenidone when compared to its control (Fig. 1e). These results suggest that high concentration of pirfenidone cause the activation of the apoptotic pathway in lung CAFs leading to cell death.

### Pirfenidone reduces TGFβ1 expression

**Fig. 2:**
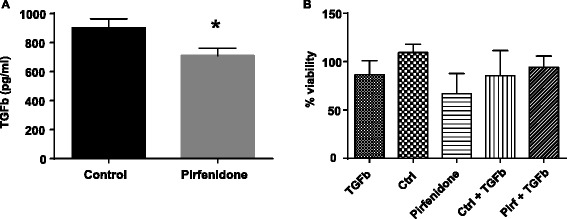
**a** TGFβ ELISA in lung CAFs treated for 72 h with pirfenidone (1.5 mg/mL). Decreased TGFβ is observed in lung CAFs in the presence of pirfenidone. **b** MTS assay showed viability of lung CAFs after 72-h treatment with pirfenidone (Pirf; 1.5 mg/mL) in the presence and absence of rhTGFβ (TGFβ, 5 ng/mL). *P < 0.05

Pirfenidone has been shown to reduce the secretion of TGFβ1 by some cell types [26–28]. To determine whether pirfenidone has the same effect on lung CAFs, we treated the cells with pirfenidone (1.5 mg/mL), and measured the amount of TGFβ1 secreted over 72 h determined by ELISA. Figure 2a shows that there was a decrease in TGFβ1 secretion when lung CAFs were treated with pirfenidone. To determine whether this had an effect on survival of the lung CAFs, a proliferation assay was performed with and without pirfenidone, in the presence and absence of recombinant human TGFβ1 (rhTGFβ1; 5 ng/mL). The addition of rhTGFβ1 did not significantly increase the proliferation in lung CAFs (Fig. 2b). Thus, addition of rhTGFβ1 does not significantly protect these cells from pirfenidone-induced cell death. Therefore, it may be that TGFβ1 is a redundant pathway by which pirfenidone induces cell death, but it is not the main pathway involved.

### Co-culture model shows increased cell death in combination treatment

**Fig. 3:**
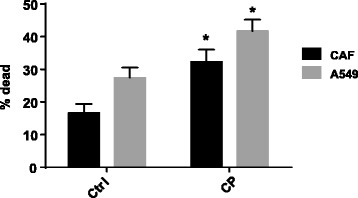
Lung CAFs and A549s were co-cultured at a 1:1 ratio. A549s were stained in to distinguish between the 2 different cells. Annexin V/PI flow cytometric analysis was performed after 48 h of treatment with cisplatin (10 μM) and pirfenidone (0.5 mg/mL). Data show an increase in dead cells (annexin V+/PI+) with combined treatment (CP) when compared to the control (Ctrl) in both cell types. *P < 0.05

To better understand whether pirfenidone has an effect in the tumor microenvironment, a two-dimensional cell culture model combining lung CAFs and NSCLC A549 cells was utilized, allowing us to observe a more physiologically relevant model of how a tumor may behave in vivo. This model enables the cross-talk between the tumor cells and the lung CAFs, which are important components of the tumor microenvironment in vitro. Furthermore, this model was designed to determine whether pirfenidone, targeting the CAFs, in combination with a known chemotherapeutic agent, cisplatin, could lead to a more robust increase in cell death. Combination studies using various concentrations of both drugs were performed. The combination that produced the best results was pirfenidone at 0.5 mg/mL and cisplatin at 10 μM. To differentiate both cell lines, for the flow cytometric analysis, the A549 cells were stained with Cell Tracker Violet® before the cells were co-cultured. We found that the combination of both drugs led to a significant increase in annexin V+/PI+ (dead) population versus control (Fig. 3) or pirfenidone or cisplatin alone (data not shown) in both the lung CAFs and the A549 cells. Thus, the combination of pirfenidone with cisplatin showed an effect on both the tumor cells and the lung CAFs.

### In vivo model of A549 and CAF leads to reduced tumor growth

**Fig. 4:**
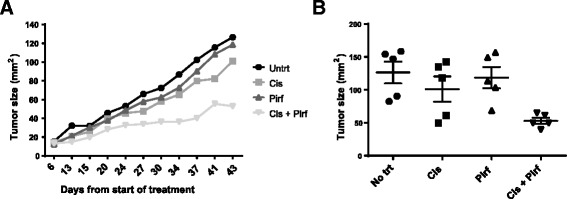
In vivo nude mouse model of lung CAFs and A549 (1:1) treated intraperitoneally with 200 mg/kg pirfenidone (Pirf; day 0–27) and 5 mg/kg cisplatin (Cis; day 0, 3, 6, 21, 24, 27) or the combination of both drugs. **a** Graph of the tumor measurements throughout the entire study. **b** Scatter plot on last day (day 43) of tumor measurements. Each group consisted of 5 mice. The experiment was done twice with similar trends. *P < 0.05

We used an in vivo model to corroborate the data obtained with the in vitro co-culture model. Nude mice were inoculated with a combination of A549 cells and lung CAF cells at a 1:1 ratio. Treatment with pirfenidone (200 mg/kg) and cisplatin (5 mg/kg) began as soon as the tumors were palpable. Pirfenidone was given daily for a total of 27 days, whereas cisplatin was given once every 3 days at the beginning of treatment and at the end of treatment. Mice were kept for an additional 16 days after treatment to observe tumor growth. Figure 4a shows the size of the tumors after the start of the treatment until the end of the study on day 43. There was no difference in the growth rate of tumors in untreated, cisplatin-treated, or pirfenidone-treated mice. However, the combination of cisplatin and pirfenidone produced a significant decrease in tumor growth (Fig. 4b).

### Pirfenidone is synergistic with cisplatin in producing cell death

**Fig. 5:**
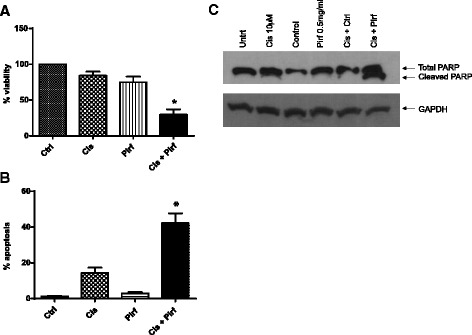
**a** MTS assay showing the viability of A549 after 72-h treatment with a low dose of cisplatin (Cis; 10 μM) and a low dose of pirfenidone (Pirf; 0.5 mg/mL). Untrt, untreated. **b** Annexin V/PI flow cytometric analysis in A549 cells after 48 h of treatment showing increased apoptosis when agents were combined (cisplatin + pirfenidone) compared with control. **c** Immunoblot analysis of PARP cleavage showing significant PARP cleavage in the combination of both drugs when compared to each drug alone. *P < 0.05

Our previous data demonstrated that the combination treatment significantly decreased cell viability and reduced tumor volume in lung CAFs and tumor cells. Next, we investigated whether the combination of pirfenidone and cisplatin had an effect on NSCLC cell lines in an in vitro model. A proliferation assay was performed using 7 NSCLC cell lines (only A549 is shown) in the presence of cisplatin (10 μM) and pirfenidone (0.5 mg/mL) for 72 h. We observed a small decrease in cell proliferation in the presence of pirfenidone alone (20 %), as well as with cisplatin alone (10 %) (Fig. 5a). As predicted, when both drugs were combined, we observed a significant decline in proliferation (60 %). Additionally, we examined 6 different NSCLC cell lines using the treatment regimen described above. Interestingly, 3 of 6 cell lines showed decreased proliferation in the combination treatment (Additional file 1). To characterize the type of cell death induced by pirfenidone and cisplatin, we performed an annexin V/PI flow cytometric analysis and showed that the combination of both drugs increased the percentage of early apoptotic cell death when compared to single-drug treatment alone (Fig. 5b). In addition, immunoblotting analysis of PARP cleavage demonstrated that there was an increase of the 89-kDa fragment (indicating apoptosis) in the combination when compared to the single-drug treatments (Fig. 5c).

**Fig. 6:**
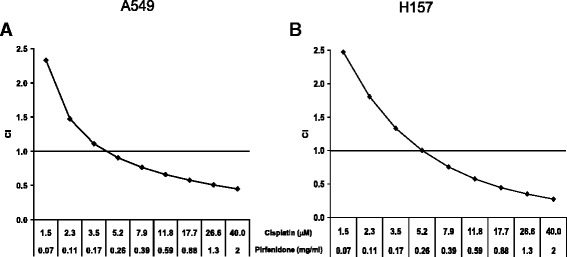
Activity of the combination of cisplatin and pirfenidone was determined using the CellTiter-Blue cell viability assay after a 72-h treatment in A549 cells (**a**) and H157 cells (**b**). Results were analyzed for synergism using the combination index (CI) method. A confidence interval of 0.1–0.3 indicated strong synergism, 0.3–0.7 indicated synergism, 0.7–0.85 indicated moderate synergism, 0.85–0.90 indicated slight synergism, and 0.90–1.10 indicated nearly additive

To determine whether the effects of the combination of pirfenidone and cisplatin were synergistic, we treated A549 and H157 cells with various concentrations of pirfenidone and cisplatin in a constant 1:50 ratio and used Calcusyn software to generate Fa-Cl plots. Figure 6a and b show the specific treatment concentrations in which the combination index values were above and below 1, indicating antagonistic and synergistic interactions, respectively. Our results show that the combination of pirfenidone and cisplatin was synergistic in both A549 and H157 NSCLC cells.

To explore the mechanism of this synergy, we examined the TGFβ signaling pathways because previous studies [24–26] have suggested that pirfenidone decreases TGFβ1 expression. We found no significant changes in proteins involved in the canonical pathway of TGFβ; specifically, cisplatin plus pirfenidone had no effect on phosphorylated Smad-2, β-catenin, and Snail (data not shown). However, we found the non-canonical TGFβ signaling pathway involving ERK and AKT was affected by cisplatin plus pirfenidone. An increase in phosphorylated ERK was observed at an early time point (15 min after treatment), whereas no changes in phosphorylated Akt were observed when pirfenidone and cisplatin were used in combination. Interestingly, a decrease in phosphorylated Akt became evident at a much later time point (48 h) when both drugs were used in combination, compared to single drug alone (Additional file 2). These studies suggest the possibility that the ERK pathway may play an integral role in the increased cell death observed in the combination treatment, since the changes were observed at a very early time point after treatment. To further explore this possibility, we inhibited the phosphorylation of ERK with the MEK inhibitor U0126. When A549 cells were treated with the inhibitor (10 μM) for 1 h before treatment was started, viability at 72 h was not influenced by cisplatin and pirfenidone (Additional file 3). These findings suggest that, although the ERK pathway is affected by cisplatin and pirfenidone, it is not the main pathway by which these drugs induce cell death in NSCLC.

## Discussion

Given the fact that CAFs actively contribute to the malignant phenotype, targeting these cells in cancer patients could produce clinical benefit. CAFs share a number of characteristics with fibroblasts that produce fibrosing diseases such as idiopathic pulmonary fibrosis. Several drugs have recently been approved for the treatment of this disease and could be repurposed as anti-cancer drugs by targeting CAFs. We demonstrated that one of these drugs, pirfenidone, could result in CAF death but only at high concentrations. However, in combination with cisplatin, lower concentrations produced CAF death. Surprisingly, we also found that pirfenidone has a direct effect on NSCLC tumor cells and therefore has the potential of functioning as an anti-cancer drug by affecting both CAFs and tumor cells.

The exact mechanism of action of pirfenidone is still unknown. Numerous studies have shown that pirfenidone plays a role in the regulation of TGFβ as well as MMP, but the precise mechanism by which the drug exerts its anti-proliferative effect in fibroblasts remains unknown. Here, we demonstrated that pirfenidone decreases TGFβ1 levels; however, the addition of rhTGFβ1 did not rescue the lung CAFs from cell death, indicating that there are other unknown pathways involved. In the tumor cells, the non-canonical TGFβ signaling pathway was affected, although functional inhibition of the pathway failed to abrogate its ability to produce cell death.

Other investigators have studied the strategy of combining pirfenidone with chemotherapy. Kozono et al. [15] showed that pirfenidone affects tumor-stromal interactions between pancreatic stellate cells and pancreatic cancer cells in vitro. In addition, they showed that the combination of pirfenidone with gemcitabine inhibited the growth of the tumors in an in vivo model of pancreatic cancer cells co-implanted with pancreatic stellate cells. Recently, Choi et al. [29] showed that the combination of sunitinib and pirfenidone led to increased radiosensitivity of NSCLC tumors in vivo. Our study is the first to show that the combination of pirfenidone and cisplatin, as demonstrated in a co-culture model as well as in an in vivo model, leads to increased cell death and tumor regression in NSCLC.

## Conclusions

Although the mechanism is as yet uncertain, the anti-fibrotic drug pirfenidone used in the treatment of idiopathic pulmonary fibrosis, when combined with cisplatin, produces CAF and surprisingly tumor cell death. This suggests a new strategy for the treatment of cancer, repurposing this drug to kill the tumor-promoting CAFs and the tumor cells themselves, which may produce a clinical benefit greater than would be expected with chemotherapy alone.

## Additional files

Additional file 1:
**MTS assay showing the viability of H358 (** **A** **), H1299 (** **B** **), H23 (** **C** **), H157 (** **D** **), H2122 (** **E** **), and PC9 (** **F** **) after 72-h treatment with a low dose of cisplatin (Cis; 10 μM) and a low dose of pirfenidone (Pirf; 0.5 mg/mL).** * *P* < 0.05. (PDF 1253 kb) Additional file 2:
**Immunoblot analysis shows an increase in phosphorylated ERK when A549 cells are treated with low doses of cisplatin (10 μM) and low doses of pirfenidone (0.5 mg/mL) at early time points (15 and 30 min;** **A** **) when compared to each drug alone.** **B:** At 48 h after treatment, there is a decrease in phosphorylated Akt when compared to each drug alone. (PDF 1211 kb) Additional file 3:
**MTS assay showing the viability of A549 cells after 72-h treatment with a low dose of cisplatin (Cis; 10 μM) and low dose of pirfenidone (Pirf; 0.5 mg/mL) in the presence and absence of the MEK inhibitor U0126 (U; 10 μM).** (PDF 3219 kb)

## Abbreviations

CAFs: cancer associated fibroblasts
CI: combination index
NSCLC: non-small cell lung cancer
rhTGFβ1: recombinant human TGFβ1
TGFβ: transforming growth factor beta
