# Supplementary figures and images for: The anti-fibrotic agent pirfenidone synergizes with cisplatin in killing tumor cells and cancer-associated fibroblasts
Source: BMC Cancer. 2016 Mar 2;16:176. doi: 10.1186/s12885-016-2162-z (PMC4776434; doi:10.1186/s12885-016-2162-z)

**A.**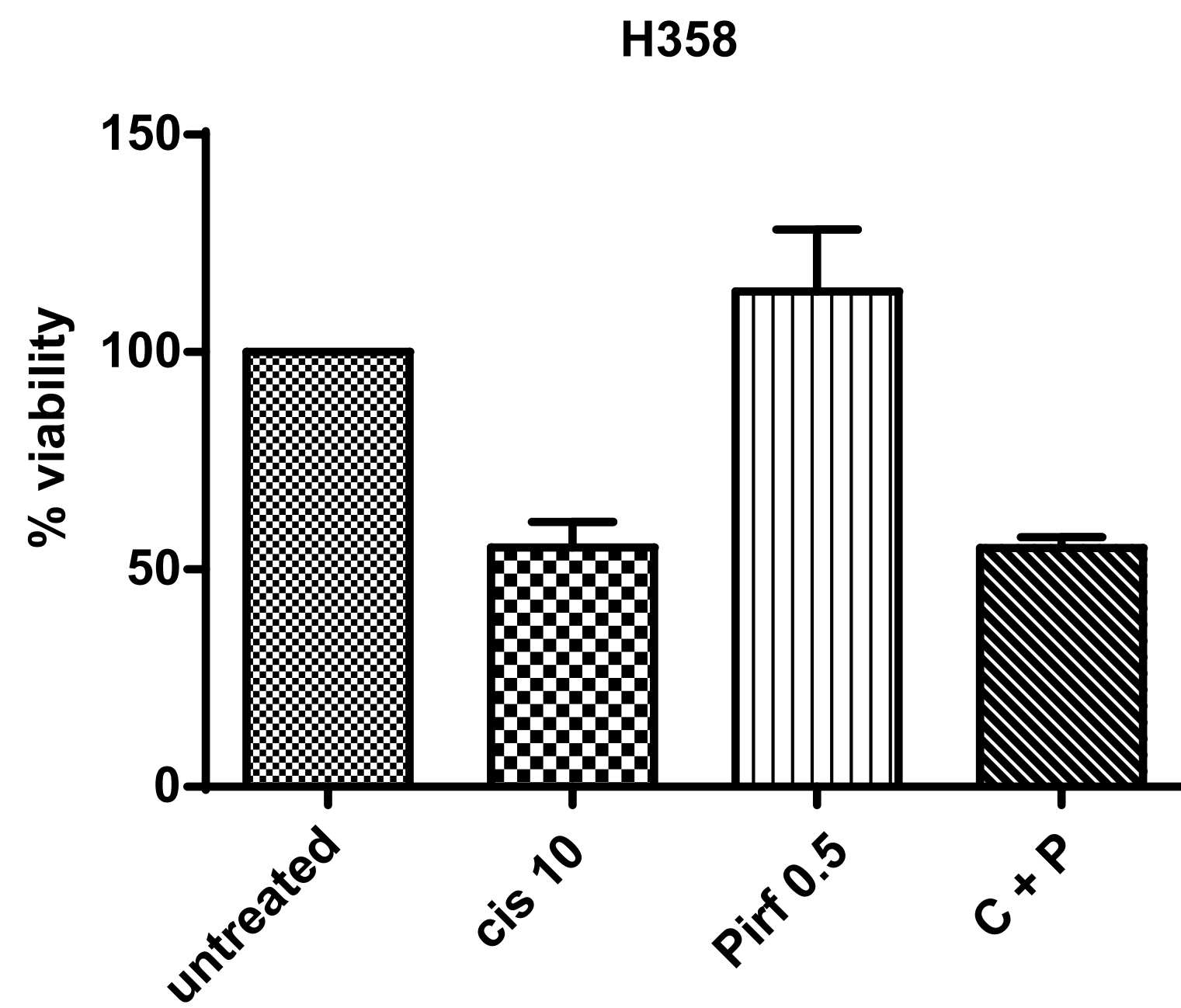**B.**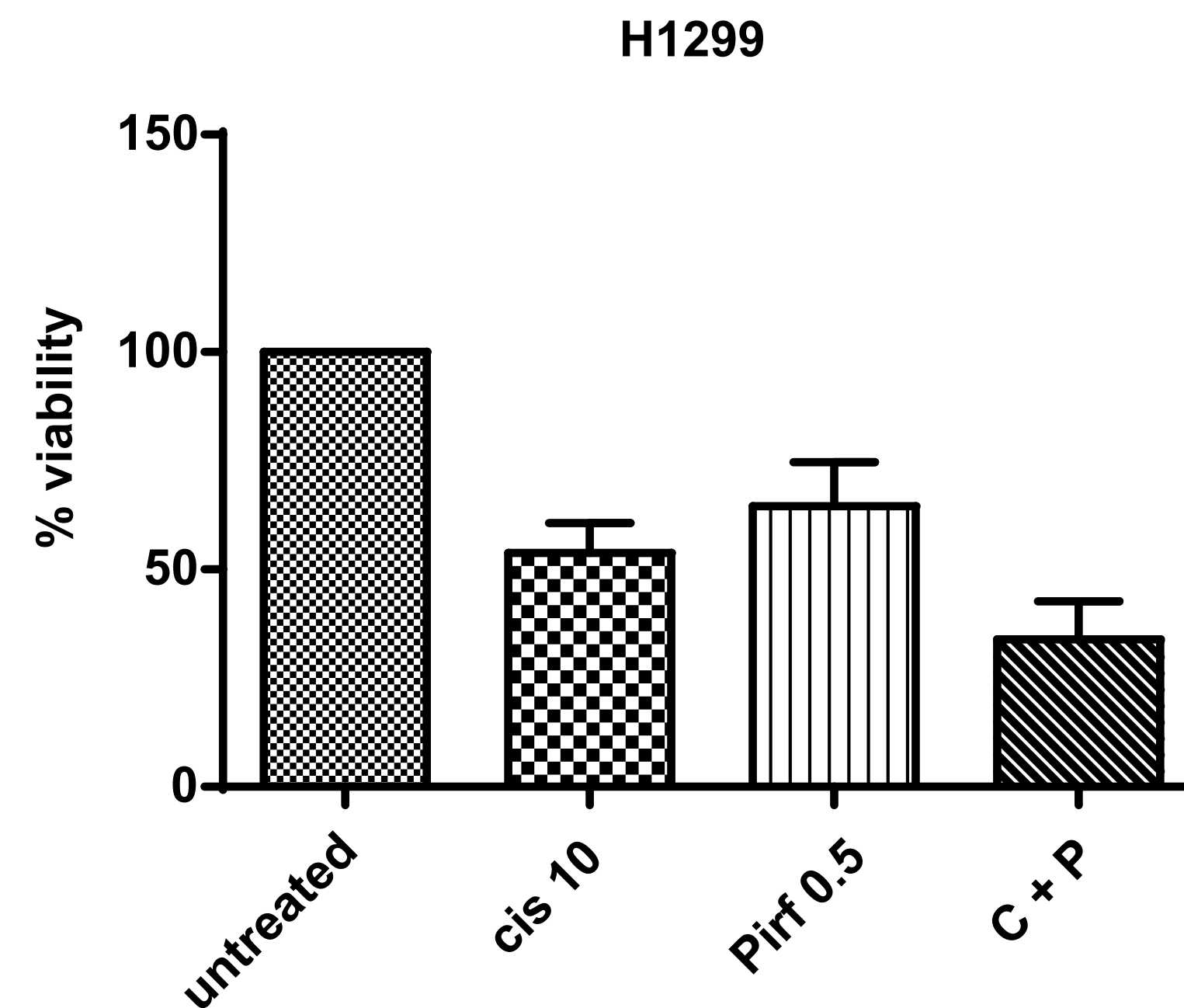**C.**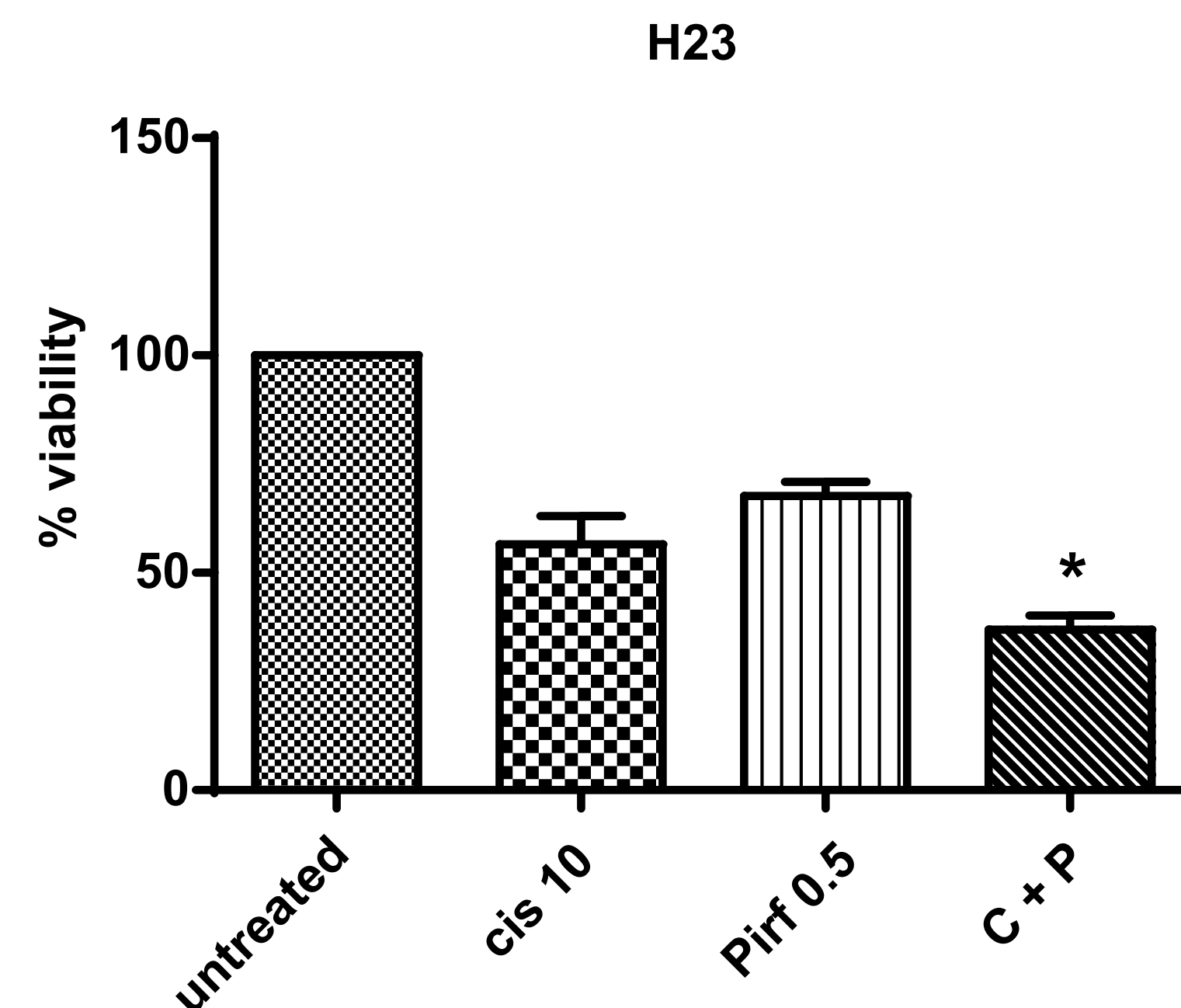**D.**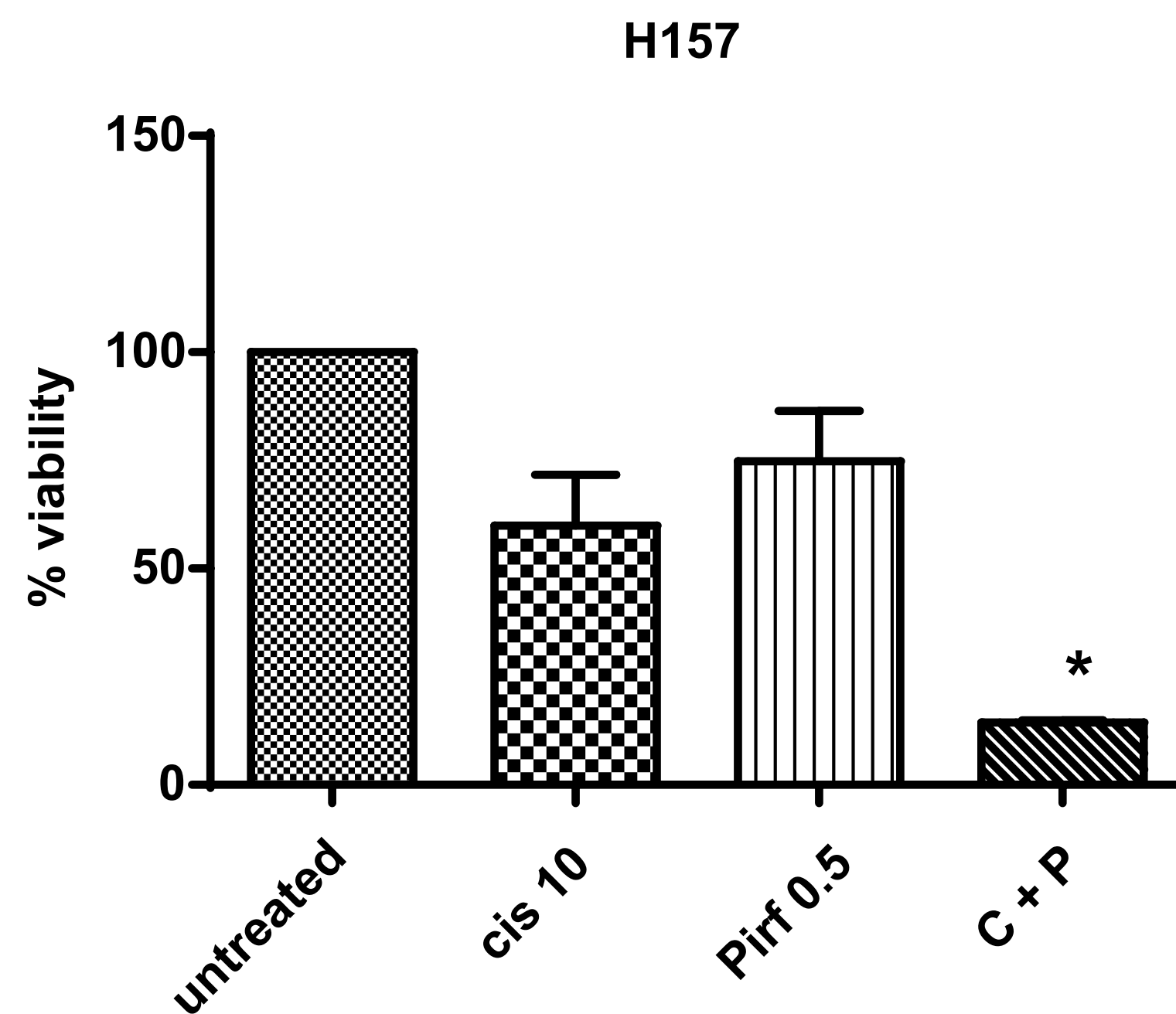**E.**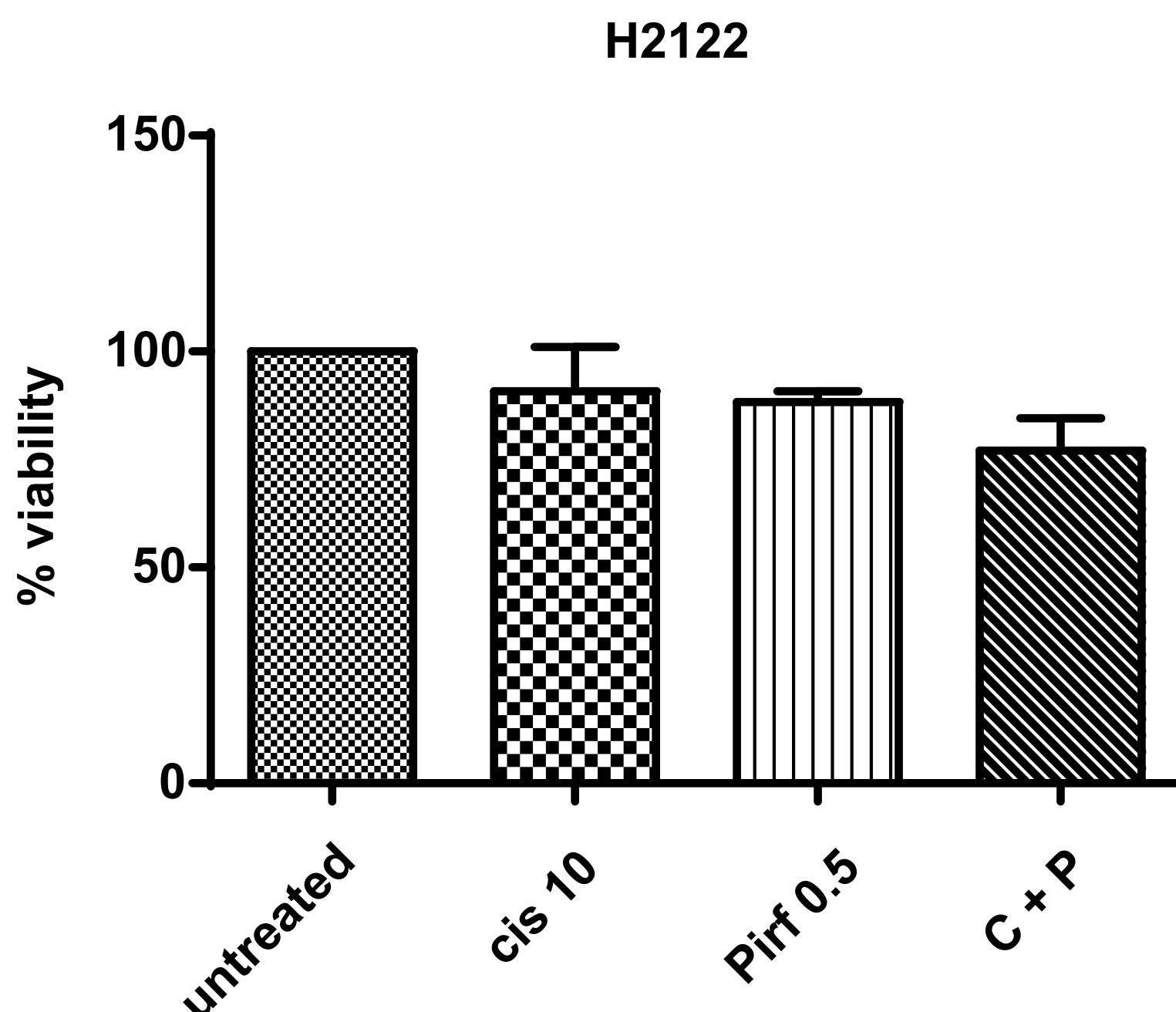**F.**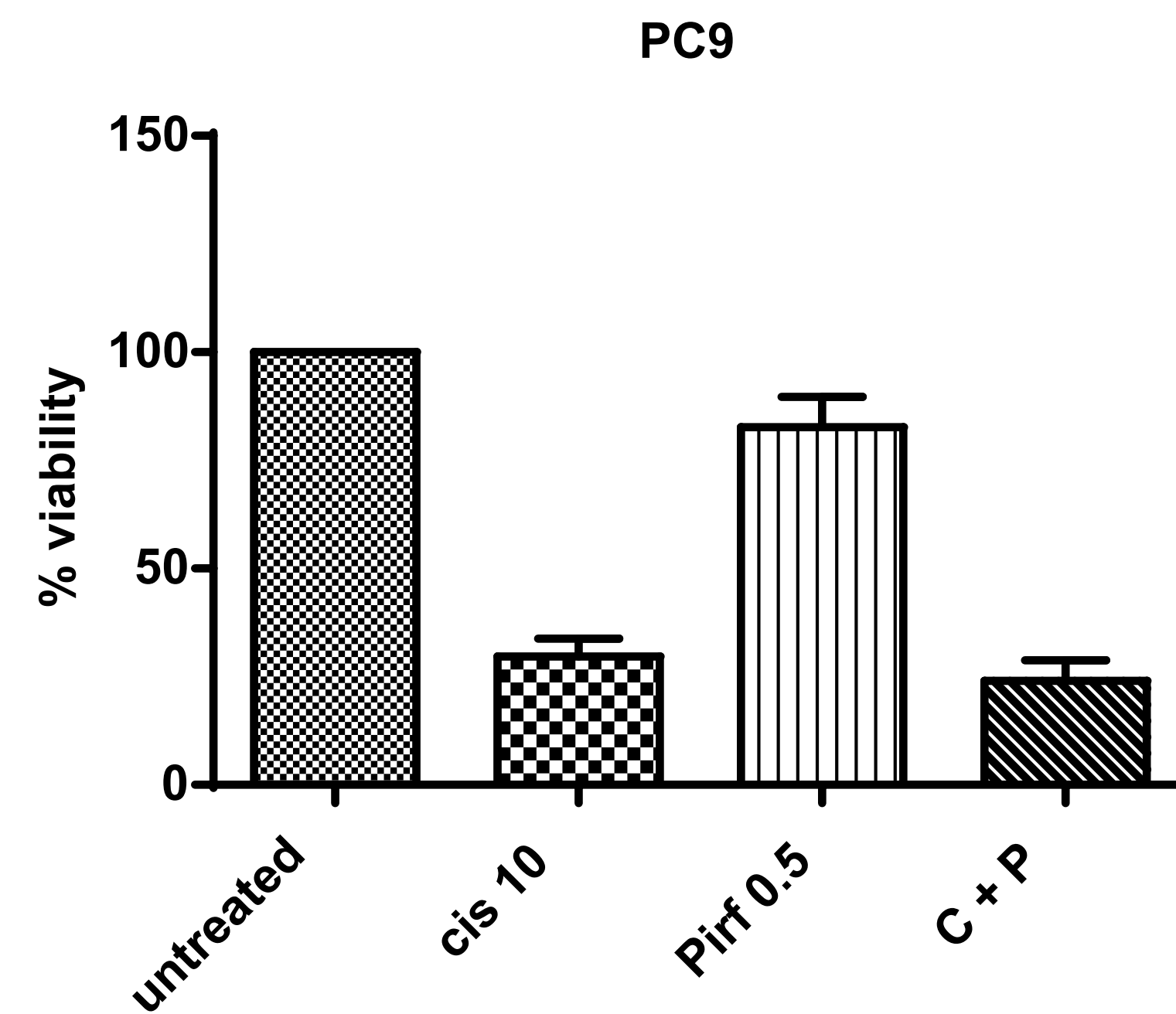

Supplement: Additional file 1: — MTS assay showing the viability of H358 ( A ), H1299 ( B ), H23 ( C ), H157 ( D ), H2122 ( E ), and PC9 ( F ) after 72-h treatment with a low dose of cisplatin (Cis; 10 μM) and a low dose of pirfenidone (Pirf; 0.5 mg/mL). * P < 0.05. (PDF 1253 kb) [file 12885_2016_2162_MOESM1_ESM.pdf]

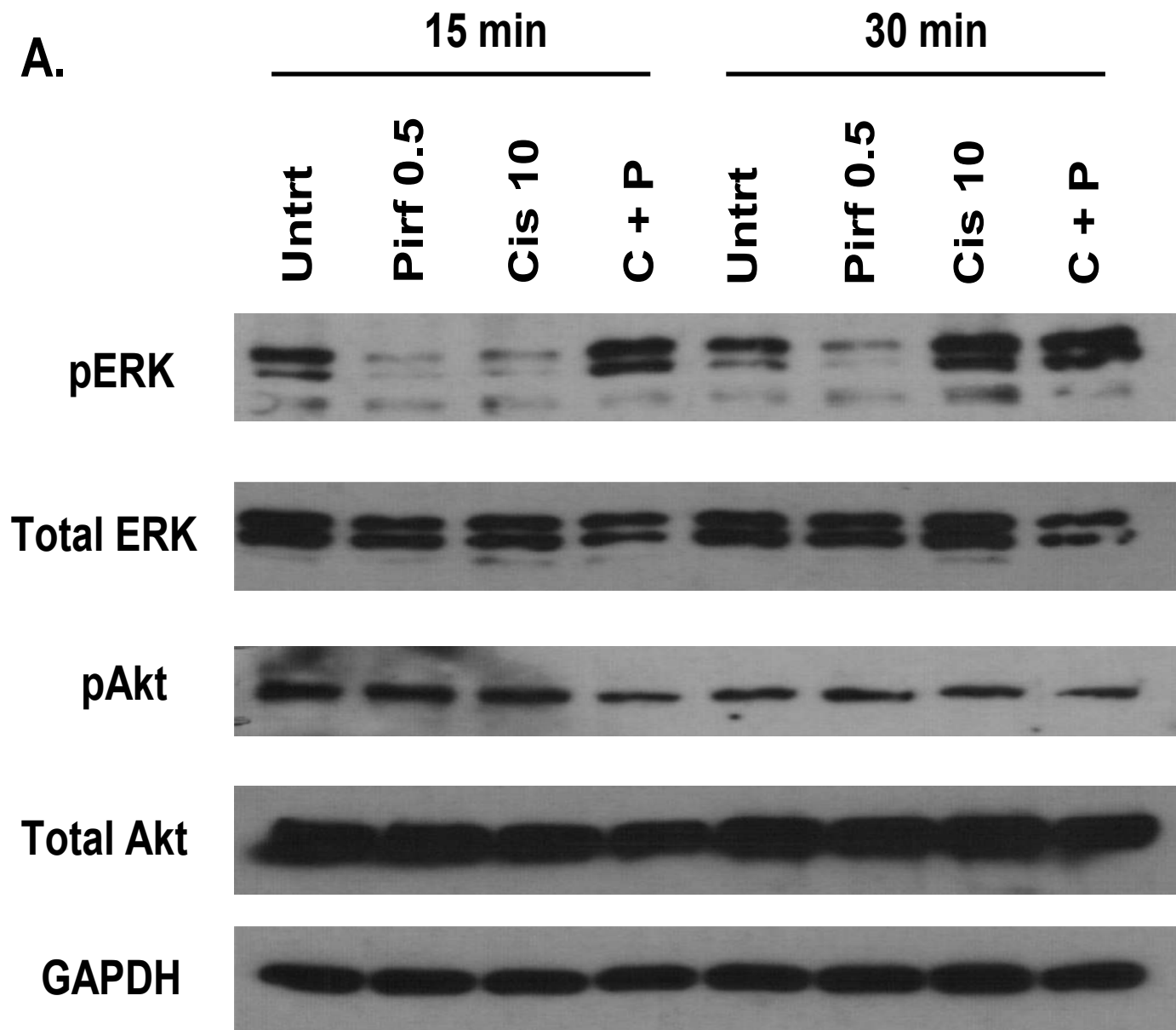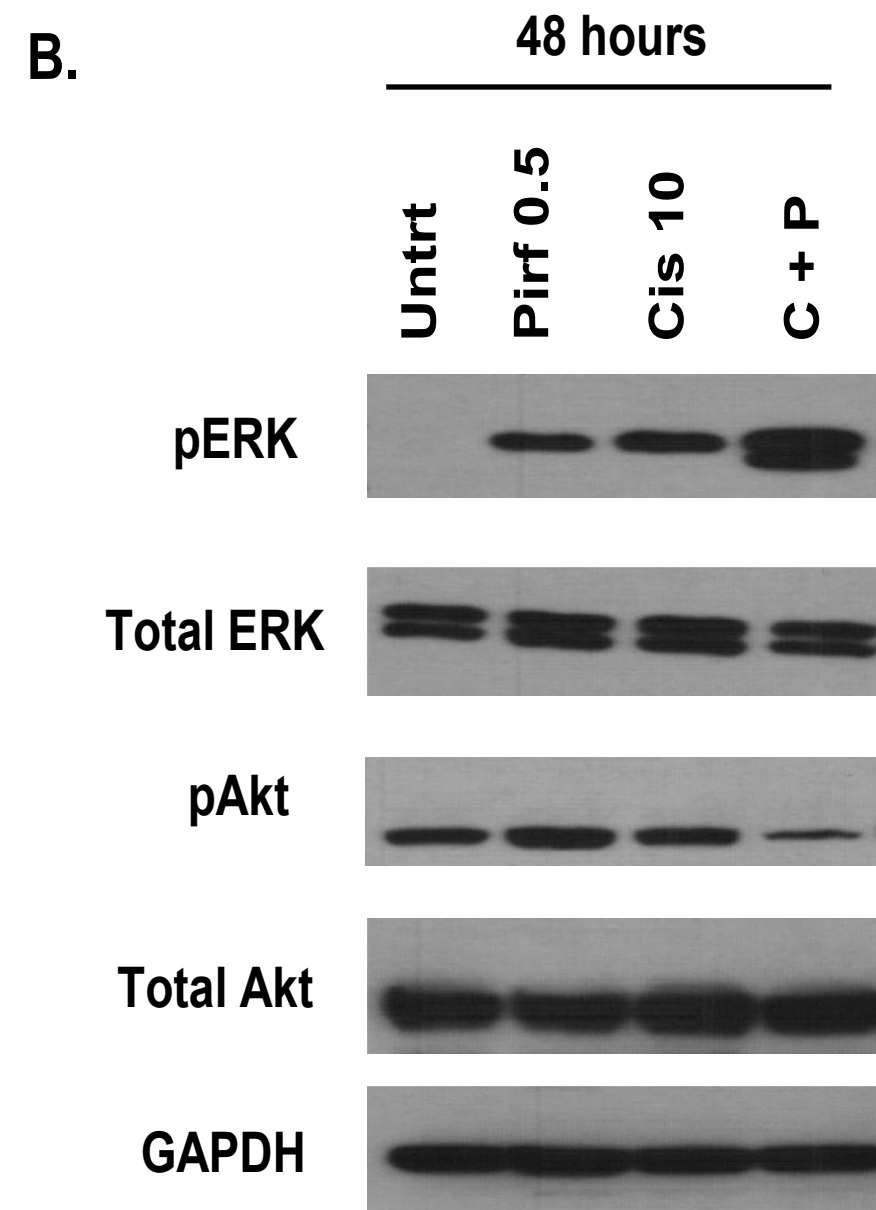

Supplement: Additional file 2: — Immunoblot analysis shows an increase in phosphorylated ERK when A549 cells are treated with low doses of cisplatin (10 μM) and low doses of pirfenidone (0.5 mg/mL) at early time points (15 and 30 min; A ) when compared to each drug alone. B: At 48 h after treatment, there is a decrease in phosphorylated Akt when compared to each drug alone. (PDF 1211 kb) [file 12885_2016_2162_MOESM2_ESM.pdf]

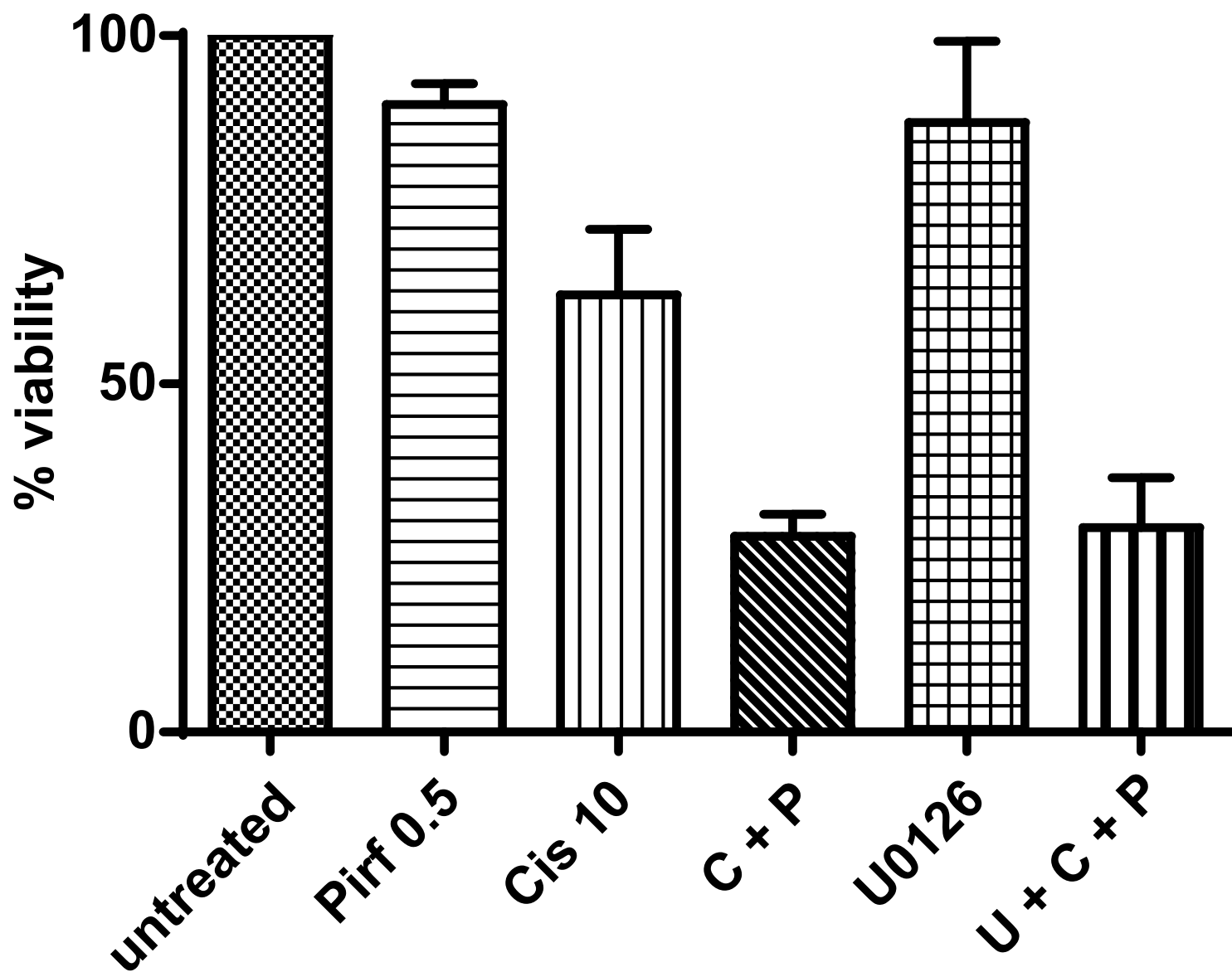

Supplement: Additional file 3: — MTS assay showing the viability of A549 cells after 72-h treatment with a low dose of cisplatin (Cis; 10 μM) and low dose of pirfenidone (Pirf; 0.5 mg/mL) in the presence and absence of the MEK inhibitor U0126 (U; 10 μM). (PDF 3219 kb) [file 12885_2016_2162_MOESM3_ESM.pdf]
